# The impact of COVID-19 pandemic on HIV care continuum in Jiangsu, China

**DOI:** 10.1186/s12879-021-06490-0

**Published:** 2021-08-07

**Authors:** Lingen Shi, Weiming Tang, Haiyang Hu, Tao Qiu, Gifty Marley, Xiaoyan Liu, Yuheng Chen, Yunting Chen, Gengfeng Fu

**Affiliations:** 1 Institute for STI and HIV Control and Prevention, Jiangsu Provincial Center for Disease Control and Prevention, No. 172 Jiangsu Road, Gulou District, Nanjing, 21009 Jiangsu China; 2 University of North Carolina Project-China, Guangzhou, 510095 China; 3 Department of Epidemiology and Health Statistics, Nanjing Medical University, Nanjing, China

**Keywords:** COVID-19, Pandemic, HIV/AIDS, Continuum, Testing, ART

## Abstract

**Background:**

The COVID-19 pandemic seriously threatens general public health services globally. This study aimed to evaluate the impact of the COVID-19 pandemic on the HIV care continuum in Jiangsu province, China.

**Methods:**

Data on newly diagnosed HIV persons for analysis were retrieved from Chinas’ web-based Comprehensive Response Information Management System (CRIMS) for HIV/AIDS from 2016 to 2020. We recorded data for the first 3 months (January to March, 2020) of strictly implementing COVID-19 measures from publicly available disease databases of the Jiangsu provincial Health Committee. We used seasonal autoregressive integrated moving average (SARIMA) and exponential smoothing in forecasting the parameters. Subgroup differences were accessed using Chi-square tests.

**Results:**

Compared to the estimated proportions, the HIV testing rates decreased by 49.0% (919,938) in the first three months of implementing COVID-19 measures. Of an estimated 1555 new HIV diagnosis expected in the same period, only 63.0% (980) new diagnoses were recorded. According to actual data recorded during the said period, 980 positively tested persons received confirmatory tests, of which 71.4% (700) were reportedly linked to care. And only 49.5% (235) out of the expected 475 newly diagnosed HIV persons received CD4 cell count testing. Meanwhile 91.6% (208) of newly diagnosed HIV persons who received CD4 count tests reportedly initiated antiretroviral therapy (ART) compared to the 227 expected. Compared to the same period from 2016 to 2019, PLWH less than 30 years old and migrants were more likely to be affected by the COVID-19 policies.

**Conclusions:**

The COVID-19 pandemic negatively impacted HIV healthcare systems in Jiangsu, China. Further measures that can counter the impact of the pandemic are needed to maintain the HIV care continuum.

**Supplementary Information:**

The online version contains supplementary material available at 10.1186/s12879-021-06490-0.

## Background

The SARS-CoV-2 virus that causes severe acute respiratory syndrome (COVID-19) has become a global pandemic since its first report in December 2019 [1–4]. Most countries have had to close borders or restrict international traffic as part of upscaled public health systems to contain transmission rates in response to the pandemic [5]. The Chinese government initiated a level one public health emergence response (PHER) in late January 2020, which restricted social contact in communities and shut down public transportation systems at the national level as part of prevention and control strategies [1]. The rules were vital for COVID-19 containment but seriously affected other public health systems and HIV care services [6]. Furthermore, HIV-related deaths and new HIV infections were expected to increase with the duration of pandemic COVID-19 due to the interruption of antiretroviral therapy (ART) [7, 8].

Located on the east coast of China, Jiangsu province was estimated to have 80.7 million people in 2019 and over 31,000 people living with HIV (PLWH) by the end of 2020 [9]. A recent estimation model showed the province to have a low HIV prevalence of 4.9 per 10,000 persons in 2020. Over time, the number of newly diagnosed HIV cases increased from 899 in 2008 to 3923 in 2020 at an annual growth rate of 13.1%. The proportion of sexual transmission also increased from 70.7% in 2008 to 99.1% in 2020, at a 2.1% growth rate yearly. Amongst newly diagnosed HIV persons, the proportion infected through homosexual behaviors accelerated from 28.0% in 2008 to 56.0% in 2020. Also, 95.1% of newly diagnosed PLWH received free CD4 count testing (a helpful marker of linkage to care system) in 2020.

To cope with the persisting stigma and low HIV awareness among the population, the Chinese government initiated the “Four free one care” policy in 2003. The policy provided free ART to all PLWH, free voluntary counseling and testing, free prevention services on mother-to-child transmission, free schooling for children orphaned or otherwise affected by HIV, and economic assistance to households of PLWH [10]. This strategy helped ease the HIV-related disease and financial burden, increased HIV screening and linkage to care, and improved the overall health of PLWH. However, some new challenges emerged over time and needed to be bridged. For example, there was a gap between the number of newly diagnosed HIV cases and the estimated number of HIV cases expected. Also, sexual transmission became the dominant transmission route [11], and the prevalence of unsafe sexual behaviors among high-risk populations such as men who have sex with men (MSM) increased [12–14].

To overcome these challenges, the Chinese government initiated the “Five expend and six strengthen” program in 2010 to expand and strengthen the HIV health care systems. The government scaled up the following nationwide: coverage and publicity of HIV education, HIV surveillance and testing, prevention of mother-to-child transmission strategies, comprehensive interventions, and ART coverage. The government also strengthened six systems that included blood donation, healthcare facilities, HIV support systems, social discrimination elimination systems, organizational leadership, and team construction [15]. The local government extended HIV testing coverage into all level two hospitals or health institutions and encouraged the promotion of community-based HIV testing, as well as HIV self-testing. However, the COVID-19 measures actively inhibited HIV services delivery. A recent study found that HIV testing rates decreased by 36.7% during the COVID-19 pandemic in China compared with the first quarter of 2019 [16]. Another study also observed a 59% decrease in the number of MSM undergoing facility-based HIV testing and a 34% reduction in ART initiation rate during the implementation of COVID-19 measures in Jiangsu province [8]. Meanwhile, PLWH faced the risk of ART interruption and disruption in chronic conditions treatment as hospitals were closed and some medical resources were re-purposed for COVID-19 cases [17–20]. Moreover, some PLWH faced pressing psychological problems such as anxiety and depression during the COVID-19 pandemic [21].

To better understand how implementing the COVID-19 related restriction measures impacted HIV care systems, this study compared predicted proportions of HIV care services along the HIV care continuum with actual numbers captured in Jiangsu province for the first three months of 2020. The specified period is when strict COVID-19 preventive measures were in effect.

## Methods

As at 2019, Jiangsu province had about 2,500 laboratories (made up of 14 confirmatory laboratories, 699 screening laboratories, and 1787 screening sites) designated for HIV testing services. These facilities report data on the number of HIV tests conducted monthly via an online system.

The web-based Comprehensive Response Information Management System (CRIMS) registration system is the real-time case reporting system for HIV care services in China. This study compared estimated data with actual data captured from the CRIMS system in Jiangsu province, China. Newly diagnosed HIV cases was retrieved for the first quarter of 2020 (January to March, 2020) when strict COVID-19 preventive measures were in effect in Jiangsu province.

### Data source

We retrieved all HIV-related data from CRIMS. All clients with confirmed positive HIV test results were linked to the CRIMS and entitled to free health care services (like physical and mental health care, referral care, CD4 testing, viral load testing once per year, ART services, amongst others). Epidemiological characteristics and clinical results of PLWH collated for reporting on CRIMS included: patient name, identification card number, age, occupation, route of infection, screening sites, CD4 cell count, ART, and so on.

We retrieved COVID-19 related case reports for Jiangsu from publicly available disease databases of the Jiangsu provincial Health Committee (http://wjw.jiangsu.gov.cn/) and merged for analysis.

### Definition of characteristics

Migratory status was determined using patients’ official household registration details. Participants whose registration was not in Jiangsu were considered immigrants [22]. Screening test sites were categorized as clinical sites, key population sites, penitentiary sites, and other sites in this study. Data on persons who sought HIV testing while accessing facility-based healthcare services (like surgery, sexual transmitted infection testing and medical examination) were "clinical sites" data. Screening test data reported from community-based sites (like voluntary counseling and testing "VCT" centers, pre-marital examination centers, entertainment venues, blood donor centers, and conscription physical examination centers) were deemed "key population sites" data. Test reports from non-healthcare institutions (like detention and prison centers) were "penitentiary sites" data. And test reports obtained via other means not specified in the system were grouped as "other sites" data.

### Prediction of the number of services to be provided during the first three months of COVID-19 measures

**Fig1:**
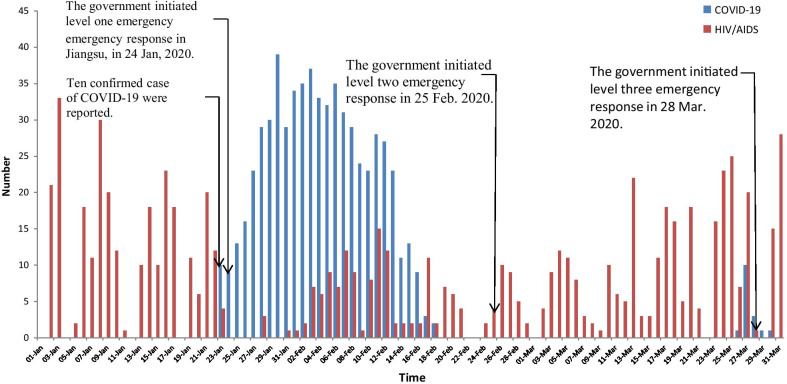
HIV/AIDS, COVID-19 daily report in Jiangsu from January to March in 2020

The HIV and COVID-19 epidemic curves were generated with data for 1^st^ January to 31^st^ March, 2020. We termed this period "the first three months of the COVID-19 measures in China" as it was the period when strict mandatory lockdowns and quarantine were in effect in many parts of China. Key dates relating to epidemic features and control policies were overlaid to aid interpretation of observed trends (Fig. 1). The autoregressive integrated moving average (ARIMA) model was used for forecasting parameters from 2016 to 2020. The seasonal ARIMA model consisted of two components (regular component and seasonal component) with six parameters (regular auto regressive (p), order of differencing (d), moving average (q), seasonal autoregressive (P), seasonal order of difference (D), and seasonal moving average (Q)). The ARIMA formula was written as ARIMA (p, d, q) (P, D, Q) s. An ARIMA model was developed using four synergistic steps, including time series stationary, model identification, parameter estimation, and diagnostic checking [23].

First, we chose a sequence diagram and white noise test to test whether the time series was stationary or not with log transformation, seasonal or non-seasonal difference. Second, we carried out the augmented Dickey-Fuller (ADF) unit root test to verify the stability of the sequence. The approximate values of p, q, P and Q were determined using Autocorrelation function (ACF) and partial autocorrelation function (PACF) graphs. Third, the actual ARIMA model was determined using parametric and residual tests. Boxs' test was used to test whether parameters were statistically significant and whether the residual was white noise. Finally, the model with the smallest AIC and BIC values was deemed the optimal model following the Akaike information criterion (AIC) and the Bayesian information criterion (BIC) [24]. If the ARIMA model was unsuitable for the data, parameters were estimated using an alternative exponential smoothing model.

Supplementary materials (Additional file 1: Fig. S2, Table S1) show all the parameters in the ARIMA and exponential smoothing model. Also, the accuracy of the forecast model formulated using the 2016–2019 data was ascertained by calculating the error rates (Additional file 1: Fig S3).

### Statistical analysis

All curves were forecasted using R packages in R software (Version 3.6.3) in a time series model for Jan 2020 to Mar 2020. Comparative analysis was based on the monthly number of reported data from January 2016 to December 2019. We also evaluated the difference in patient characteristics between the first quarter of 2020 and cumulative proportions for the first quarters of 2016 to 2019. P-value of a = 0.05 was deemed statistically significant.

### Ethical statement

Participants provided written informed consent before the interview and blood collection. Before data analysis, we exempted patient identification information like name, ID number, phone number, and home address. The Institutional Review Board of the National Center for AIDS/STD Control and Prevention (NCAIDS) and the Center for Disease Control and Prevention (CDC) in China reviewed and approved the study process. The study process and contents followed relevant guidelines.

## Results

Our reported findings follow the structure of the HIV care continuum from testing to ART initiation due to the short period.

### Testing

**Fig. 2:**
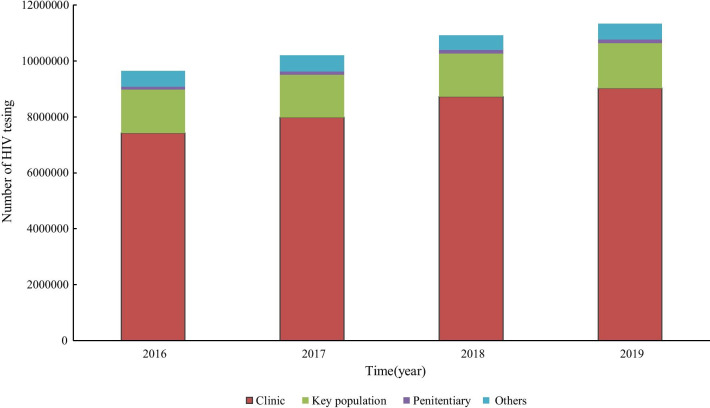
Trend of HIV screening test in four categorized groups from 2016 to 2019

**Fig. 3:**
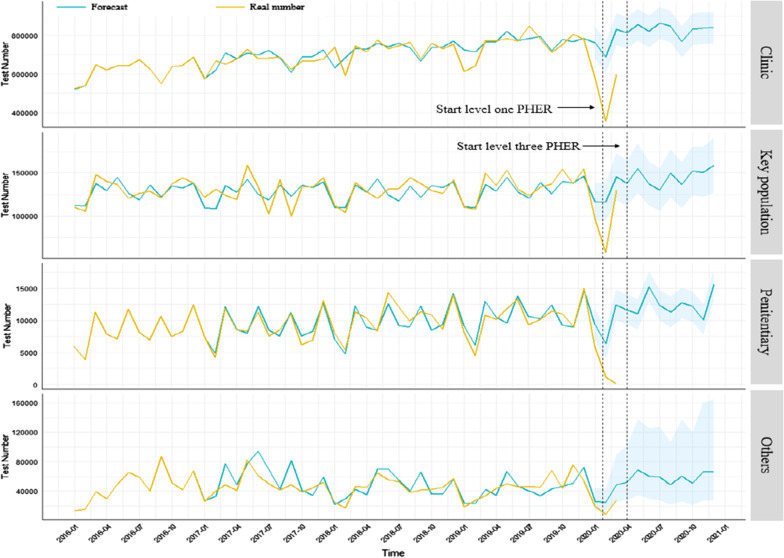
Trend of forecast and real number of HIV screening tests in four categorized groups during 2016 to 2020

The actual number of screening tests reported each year for Jiangsu province accelerated from 9,644,750 in 2016 to 11,326,388 in 2019. The majority of HIV screening tests were reported in clinical sites, and the annual proportion increased by 1.1% from 77.0% in 2016 to 79.6% in 2019 (Fig. 2). The estimated curve of screening tests was quite similar to the actual number of screening tests recorded from 2016 to 2019 (Fig. 3). For the first period of COVID-19 measures, the estimated number of HIV screening tests expected was 2,795,623. However, the actual number of HIV screening tests recorded was 1,875,685 for the period. That signified a 49.0% (919,938) decrease in HIV screening tests during the first period of COVID-19 measures.

During the same period, the actual number of HIV-positive tests recorded was 2401, of which only 40.8% (980) received confirmatory tests. That was a significant decrease compared to the 51.5% that received confirmatory tests in the same quarter from 2016 to 2019 (Additional file 1: Fig S2).

### Linkage to care

**Fig. 4:**
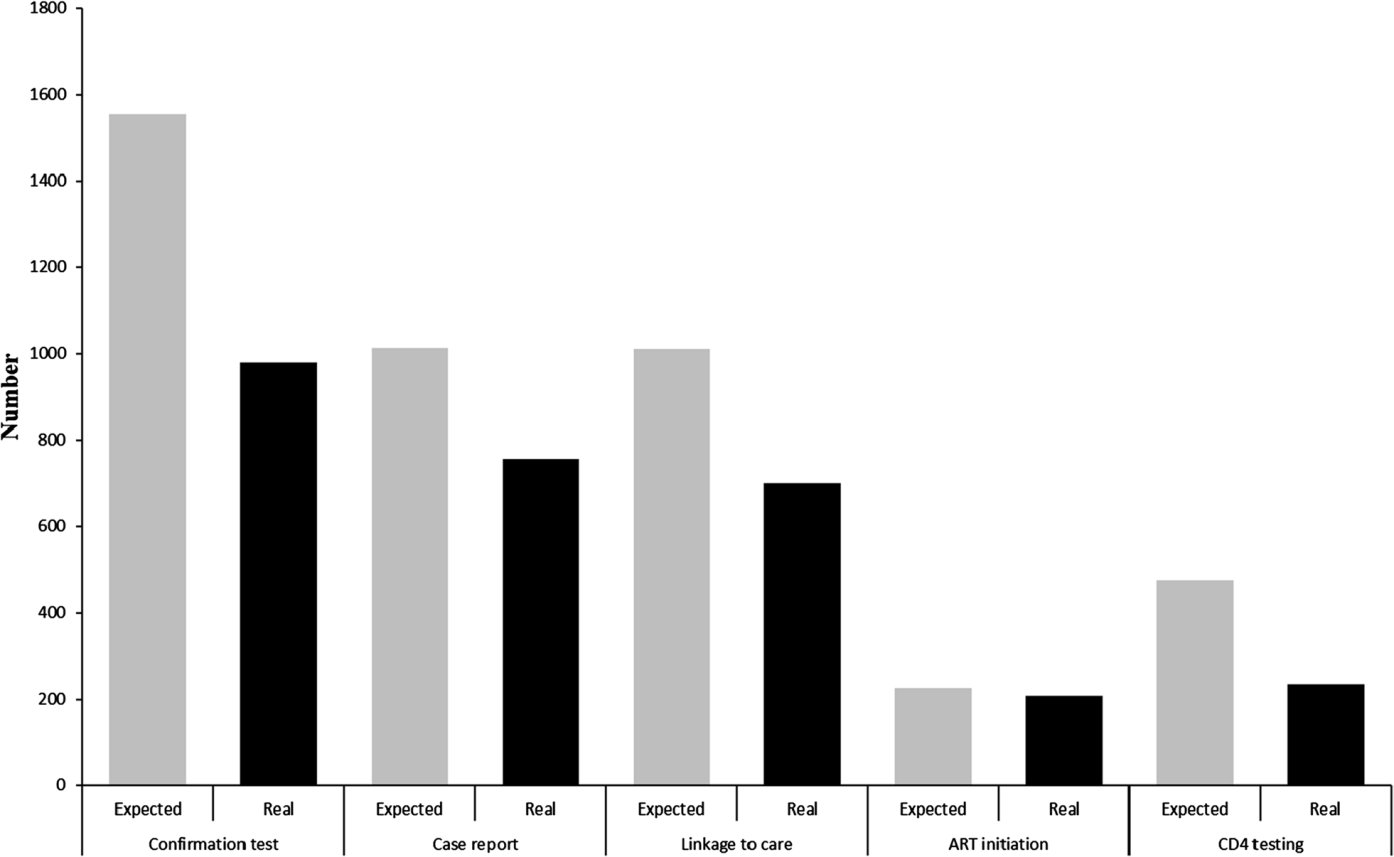
Cascade of linkage to healthcare success rates during COVID-19 era in Jiangsu, China, 2020

Based on the estimation model, 3789 positive screening tests records with a 0.2%positive test proportion were expected during the COVID-19 measures. However, only 2401 positive tests were recorded with a 0.1% positive test proportion. That showed a 36.6% decrease in the number of positive screening tests for the period. There was also a significant difference in reactive test proportions between the estimated and actual numbers (P < 0.001). Moreover, the number of confirmatory tests recorded was fewer by 37.0% when compared to the estimated 1555 confirmatory tests expected. Of all 980 confirmed cases recorded, only 77.2% were reported on CRIMS, and 71.4% were reportedly linked to care. Also, only 49.5% of the estimated 475 newly diagnosed clients received CD4 count tests (Fig. 4, Additional file 1: Fig S1). Meanwhile, the proportion that received CD4 count tests decreased significantly by 10.7% compared to the first quarter during 2016 to 2019 (P < 0.001) (Table 1). About 28.6% of all newly diagnosed PLWH during the specified time under consideration became lost-to-follow-up.

**Table 1:** Characteristics of newly diagnosed HIV cases in the first quarters of 2016 to 2020

Characteristics	2016–2019 Q1	2020 Q1	Chi square (P value)
Age group			
< 30	1400 (34.7)	215 (28.7)	15.596 (0.000)
30–49	1526 (37.8)	281 (37.5)	
≥ 50	1108 (27.5)	253 (33.8)	
Gender			
Male	3518 (87.2)	665 (88.8)	1.291 (0.256)
Female	516 (12.8)	84 (11.2)	
Education			
Primary and below	598 (14.8)	162 (21.6)	22.841 (0.000)
Junior middle school	1318 (32.7)	238 (31.8)	
High school and above	2118 (52.5)	349 (46.6)	
Marital status			
Single	1554 (38.5)	260 (34.7)	4.569 (0.102)
Married	1918 (47.6)	370 (49.4)	
Divorced or widow	562 (13.9)	119 (15.9)	
Migration			
Yes	1677 (41.6)	280 (37.4)	4.413 (0.036)
No	2357 (58.4)	469 (62.6)	
Routine of transmit			
Homosexual	2212 (54.8)	392 (52.3)	2.544 (0.279)
Heterosexual	1700 (42.1)	338 (45.1)	
Others	122 (3.0)	19 (2.5)	
CD4 test			
Yes	1733 (43.0)	242 (32.3)	29.119 (0.000)
No	2301 (57.0)	507 (67.7)	
ART			
Yes	704 (17.5)	208 (21.0)	42.920 (0.000)
No	3330 (82.6)	541 (79.0)	

### ART initiation

Compared with the actual number of newly diagnosed PLWH 700 were linked to care, 29.7% (208) initiated ART immediately. However, when considering the predicted number of ART initiation 227, 91.6% were reported to have initiated ART (Fig. 4, Additional file 1: Fig S1).

### Characteristics of newly diagnosed PLWH during COVID-19

The number of newly diagnosed PLWH less than 30 years old decreased by 6.0% in the first quarter of 2020 compared with proportions recorded in the first quarter of 2016 to 2019. However, the number of newly diagnosed PLWH aged over 50 years increased by 6.3% during the same period (P < 0.001). The number of newly diagnosed PLWH among migrants decreased by 4.2% during the period under review (P = 0.032) (Table 1).

## Discussion

The COVID-19 outbreak has had a series of impacts on HIV health care systems in Jiangsu, China. The number of reported HIV screening tests decreased by 49.0% during the COVID-19 pandemic days compared to the expected estimated data. Almost 60% of people who tested positive during HIV screening tests within that period received confirmatory tests. Moreover, only 80% of confirmed cases were registered in the healthcare system. However, most of the newly diagnosed PLWH enrolled into care did not receive timely health care services. Only an estimated one-third of all newly diagnosed PLWH received CD4 cell count tests, and less than 30% of them initiated ART during the period.

The number of HIV screening laboratories increased rapidly in hospital facilities being part of the response to achieving the global “90–90–90” targets. Therefore, hospital-based screening tests were dominant and accounted for 79.6% of all reported screening tests in 2019 for Jiangsu. However, during the COVID-9 outbreak, the preponderance of hospitals had to shut down clinical services or shifted the majority of their health care resources to complement COVID-19 control efforts [18, 25]. The level one PHER measures halted HIV-related services and decreased the chances for people to check their HIV status during the period. The local CDC offices that also played an essential role in HIV services delivery had to mobilize and relocate all health resources to cope with the COVID-19 outbreak. That also led to the cancellation of some scheduled HIV screening and confirmatory testing during the period under review. Even for institutions that could still provide HIV-related care, the lack of transport due to quarantine and social distancing policies hindered access to the services. In addition, some people shied away from getting routine HIV testing, especially in the hospital during the period as they feared facing COVID-19 related stigma and discrimination [21, 26]. It is also possible that HIV testing rates decreased due to reduced sexual risk behaviors as the majority of people (especially members of high-risk populations) spent most of the time at home during the COVID-19 measures. This explanation is possible as an online survey conducted in China showed that 44% of people reported a decrease in sexual partners around that time [27]. In another study, 45% of MSM reported a decline in their quantity of sexual partners during the COVID-19 measures period in Jiangsu, China [unpublished data]. With the decreased opportunities to have sex, participants may have perceived a less risk of exposure and were less likely to get HIV testing during the period.

PLWH already diagnosed as HIV positive could access free HIV-related health care services (like face-to-face counseling and free ART initiation) in China with the help of service providers (such as healthcare workers, CDC staff, or community-based organization "CBO" staffs) when requested. However, people with reactive results in the screening test phase faced a challenge in accessing confirmatory tests due to lockdowns during the COVID-19 phase [25]. Our findings also showed that about 28.6% of newly diagnosed PLWH became lost to follow-up or failed to get enrolled into care during the COVID-19 pandemic. In addition, the policy on quarantine challenged the timely initiation of ART as only about 20% of newly diagnosed PLWH started their ART during the COVID-19 epidemic. Until 2019, only 40 hospitals could provide ART and needed to meet the needs of more than 20,000 PLWH in Jiangsu province. In addition, PLWH enrolled in care faced potential discontinued of ART due to movement restriction policies implemented during the COVID-19 outbreak. An online survey conducted during the level one PHER found 32.6% of PLWH to be at risk of ART discontinuation soon [4]. In another survey conducted by telephone, 19.1% of PLHW did not get medicine supply in China during the COVID-19 outbreak [1]. Furthermore, some PLWH believed hospital centers to be risky sites for SARS-Cov-2 infection and hence, resisted the urge to receive services at facilities during the outbreak period [26].

Our findings showed how the COVID-19 pandemic affected the whole HIV healthcare system. Being unable to maintain HIV care during the COVID-19 pandemic was a significant hindrance to achieving the “90–90–90” goal. With the temporary discontinuation of public HIV screening systems, CBOs represented one option to bridging the gap in maintaining HIV care services [28–31]. In addition, HIV self-testing (HIVST) is an innovative approach to providing access to HIV testing safely while maintaining social distancing. Therefore, promoting HIVST will help expand the network of HIV testing especially among key populations in this era [32, 33]. Besides, HIVST affords convenience and privacy which can help expand the coverage of HIV testing to reach persons hindered from accessing facility-based testing [34, 35]. Also, using a “one-stop service” centers approach may reduce the duration between test screening and confirmatory testing [36]. Hence, adopting “one-stop service” centers could also help improve linkage to care success rates. Also, the Chinese NCAIDS guaranteed free antiviral drugs in some selected treatment management agencies to resolve the challenges that resulted from the discontinued ART services. Therefore, PLWH could refill antiviral medications at the nearest selected locations or by post to continue ART.

Our study had some limitations. First, there may have been some delays in entering data entry onto the HIV healthcare system due to the COVID-19 outbreak. That may have affected our data and results. Secondly, as most patients self-reported on their high-risk behaviors, some patients (especially those who had homosexual contacts) may have misreported behaviors due to fear of stigma or misunderstanding, especially amongst patients who have had. In addition, we could not evaluate the clinical outcome on viral load suppression since this cross-sectional study was only evaluating data on PLWH newly diagnosed during the three months of strictly state-enforced COVID-19 preventive measures. Despite these limitations, our study showed that the outbreak of COVID-19 and the policy on quarantine and lockdowns negatively effect the HIV healthcare system.

## Conclusions

In conclusion, the COVID-19 outbreak and its containment protocols like quarantine and lockdown measures crippled the HIV care continuum systems in Jiangsu province. Therefore, the local government should consider extending HIV-testing coverage by encouraging HIVST and community-based testing. Also, there is a need for a more flexible policy for ART initiation in the face of this new challenge.

## Supplementary Information

**Additional file 1: Fig S1.** Trend of forecast and real number of HIV from screen positive to ART during 2016 to 2020. **Fig S2.** Seasonal variation, autocorrelation function and partial autocorrelation function for the forecasting data. **Fig S3.** The error rate in the cascade flow in HIV health care system during 2016 to 2019. **Table S1**. Models parameters of the forecasting.

## Data Availability

COVID-19 related case reports for Jiangsu were retrieved from publicly available disease databases of Jiangsu provincial Health Committee (http://wjw.jiangsu.gov.cn/). The other datasets used in this study are available from the corresponding author on reasonable request.

## Abbreviations

SARS-CoV-2: Severe acute respiratory syndrome coronavirus 2
COVID-19: Coronavirus Disease 2019
PHER: Public health emergence response
ART: Antiretroviral therapy
PLWH: People living with HIV
CRIMS: Comprehensive Response Information Management System
ARIMA: Autoregressive integrated moving average
ADF: Augmented Dickey-Fuller
ACF: Autocorrelation function
PACF: Partial autocorrelation
AIC: Akaike information criterion
BIC: Bayesian information criterion
NCAIDS: National Center for AIDS/STD Control and Prevention
CDC: Center for Disease Control and Prevention
CBO: Community based organization
HIVST: HIV self-testing

## References

[1] Lu H, Stratton CW, Tang YW (2020). Outbreak of pneumonia of unknown etiology in Wuhan, China: the mystery and the miracle. J Med Virol.

[2] World Health Organization. Clinical management of severe acute respiratory infection when novel coronavirus (2019-nCOV) infection is suspected. 2020. https://apps.who.int/iris/handle/10665/330893. Accessed 20 May 2020.

[3] Joint United Nations Programme on HIV/AIDS (UNAIDS). Fact sheet: World AIDS Day 2019-global HIV statistics. 2019. https://www.unaids.org/en/resources/fact-sheet. Accessed 10 May 2020.

[4] Guo W, Weng HL, Bai H, Liu J, Wei XN, Zhou K, Sande A (2020). Quick community survey on the impact of COVID-19 outbreak for the healthcare of people living with HIV. Chin J Epidemiol.

[5] Zhu N, Zhang D, Wang W, Li X, Yang B, Song J (2020). A novel coronavirus from patients with pneumonia in China, 2019. N Engl J Med.

[6] Jiang H, Zhou Y, Tang W (2020). Maintaining HIV care during the COVID-19 pandemic. Lancet HIV.

[7] Hogan AB, Jewell BL, Sherrard-Smith E, Vesga JF, Watson OJ, Whittaker C (2020). Potential impact of the COVID-19 pandemic on HIV, tuberculosis, and malaria in low-income and middle-income countries: a modelling study. Lancet Glob Health.

[8] Booton RD, Fu G, MacGregor L, Li J, Ong JJ, Tucker JD (2020). Estimating the impact of disruptions due to COVID-19 on HIV transmission and control among men who have sex with men in China. medRxiv.

[9] Jiangsu commission of health. The progression of HIV/AIDS prevention and control in Jiangsu. http://wjw.jiangsu.gov.cn/art/2020/11/30/art_55460_9585843.html. Accessed 29 April 2021.

[10] Sun X, Lu F, Wu Z (2010). Evolution of information-driven HIV/AIDS policies in China. Int J Epidemiol..

[11] National Center for AIDS/STD Control and Prevention, China CDC Annual Report of China National HIV/STD/HCV Comprehensive Prevention and Treatment Programs in 2018. Beijing: National Center for AIDS/STD control and prevention, China CDC; 2019.

[12] Zhu Z, Yan H, Wu S (2019). Trends in HIV prevalence and risk behaviours among men who have sex with men from 2013 to 2017 in Nanjing, China: a consecutive cross-sectional survey. BMJ Open..

[13] Guo Y, Xu X, Fu G (2017). Risk behaviours and prevalences of HIV and sexually transmitted infections among female sex workers in various venues in Changzhou, China. Int J STD AIDS.

[14] Zhao P, Tang W, Cheng H (2020). Uptake of provider-initiated HIV and syphilis testing among heterosexual STD clinic patients in Guangdong, China: results from a cross-sectional study. BMJ Open..

[15] The Central People’s Government of the People’s Republic of China. Strengthen the prevention and control of AIDS. 2011. http://www.gov.cn/zwgk/2011-02/16/content_1804536.htm. Accessed 9 May 2021.

[16] Jiang HB, Shi HB, Feng HW (2020). Impact of COVID-19 epidemic on AIDS prevention and control in Ningbo, Zhejiang. Dis Surveillance.

[17] Chenneville T, Gabbidon K, Hanson P, Holyfield C (2020). Estimating the impact of disruptions due to COVID-19 on HIV transmission and control among men who have sex with men in China. MedRxiv.

[18] Ji T, Chen HL, Xu J, Wu LN, Li JJ, Chen K (2020). Lockdown contained the spread of 2019 novel coronavirus disease in Huangshi city, China: early epidemiological findings. Clin Infec Dis.

[19] Reza-Paul S, Lazarus L, Haldar P, Reza Paul M, Lakshmi B, Ramaiah M (2020). Community action for people with HIV and sex workers during the COVID-19 pandemic in India. WHO South East Asia J Public Health.

[20] CNN. People living with HIV in Wuhan struggle to find medicine during coronavirus outbreak (2021-04-27). https://www.cnn.com/2020/03/13/health/coronavirus-china-hiv-wuhan-intl-hnk/index.html.

[21] Sun S, Hou J, Chen Y, Lu Y, Brown L, Operario D (2020). Challenges to HIV care and psychological health during the COVID-19 pandemic among people living with HIV in China. AIDS Behav.

[22] Wu J, Wu H, Li P, Lu C (2016). HIV/STIs risks between migrant MSM and local MSM: a cross-sectional comparison study in China. PeerJ..

[23] Zheng YL, Zhang LP, Zhang XL, Wang K, Zheng YJ (2015). Forecast model analysis for the morbidity of tuberculosis in Xinjiang. China PLoS One.

[24] Cao S, Wang F, Tam W, Tse LA, Kim JH, Liu J, Lu Z (2013). A hybrid seasonal prediction model for tuberculosis incidence in China. BMC Med Inform Decis Mak.

[25] Lau H, Khosrawipour V, Kocbach P, Mikolajczyk A, Schubert J, Bania J, Khosrawipour T (2020). The positive impact of lockdown in Wuhan on containing the COVID-19 outbreak in China. J Travel Med.

[26] He J, He L, Zhou W, Nie X, He M (2020). Discrimination and social exclusion in the outbreak of COVID-19. Int Environ Res Public Health.

[27] Li W, Li G, Xin C, Wang Y, Yang S (2020). Challenges in the practice of sexual medicine in the time of COVID-19 in China. J Sex Med.

[28] Joint United Nations Programme on HIV/AIDS (UNAIDS). UNAIDS and China working together during the COVID-19 outbreak to ensure that people living with HIV continue to get treatment. 2020. https://www.unaids.org/en/resources/presscentre/pressreleaseandstatementarchive/2020/february/20200218_china_covid19. Accessed 20 May 2020.

[29] Zhang W, Hu Q, Tang W, Jin X, Mao X, Lu T (2020). HIV self-testing programs to men who have sex with men delivered by social media key opinion leaders and community-based organizations are both effective and complementary: a national pragmatic study in China. J Acquire Immune Defic Syndr.

[30] Shapatava E, Rios A, Shelley G, Milan J, Smith S, Uhl G (2018). Community-Based Organization adaptations to the changing HIV prevention and care landscape in the southern United States. AIDS Educ Prev.

[31] DeShields RD, Lucas JP, Turner M, Amola K, Hunter V, Lykes S (2020). Building partnerships and stakeholder relationships for HIV prevention: longitudinal cohort study focuses on community engagement. Prog Community Health Partnersh.

[32] Pettifor A, Lippman SA, Kimaru L, Haber N, Mayakayaka Z, Selin A (2020). HIV self-testing among young women in rural south Africa: a randomized controlled trial comparing clinic-based HIV testing to the choice of either clinic testing or HIV self-testing with secondary distribution to peers and partners. E Clinical Medicine.

[33] Katz DA, Golden MR, Hughes JP, Farquhar C, Stekler JD (2018). HIV self-testing increases HIV testing frequency in high-risk men who have sex with men: a randomized controlled trial. J Acquir Immune Defic Syndr.

[34] Mathews A, Farley S, Conserve DF, Knight K, Le'Marus A, Blumberg M (2020). “Meet people where they are”: a qualitative study of community barriers and facilitators to HIV testing and HIV self-testing among African Americans in urban and rural areas in North Carolina. BMC Public Health.

[35] Harichund C, Karim QA, Kunene P, Simelane S, Moshabela M (2019). HIV self-testing as part of a differentiated HIV testing approach: exploring urban and rural adult experiences from Kwazulu-Natal, South Africa using a cross-over study design. BMC Public Health.

[36] Zhao M, Wang X, Qiu H, Xie N, Zhang X, Wang L, et al. The effectiveness of one-stop service model on adherence in MSM infected with HIV/AID: a pilot study. Chin J AIDS STD 2013. CNKI: SUN: XBYA.0.2013-08-015

