# Supplementary material for: The impact of COVID-19 pandemic on HIV care continuum in Jiangsu, China
Source: BMC Infect Dis. 2021 Aug 7;21:768. doi: 10.1186/s12879-021-06490-0 (PMC8346346; doi:10.1186/s12879-021-06490-0)
Supplement: Supplementary file 1 — Additional file 1: Fig S1. Trend of forecast and real number of HIV from screen positive to ART during 2016 to 2020. Fig S2. Seasonal variation, autocorrelation function and partial autocorrelation function for the forecasting data. Fig S3. The error rate in the cascade flow in HIV health care system during 2016 to 2019. Table S1. Models parameters of the forecasting. [file 12879_2021_6490_MOESM1_ESM.doc]

**The impact of COVID-19 pandemic on HIV care continuum in Jiangsu, China**

**Lingen Shi, Weiming Tang, Haiyang Hu, Tao Qiu, Gifty Marley, Xiaoyan Liu, Yuheng Chen, Yunting Chen, Gengfeng Fu**


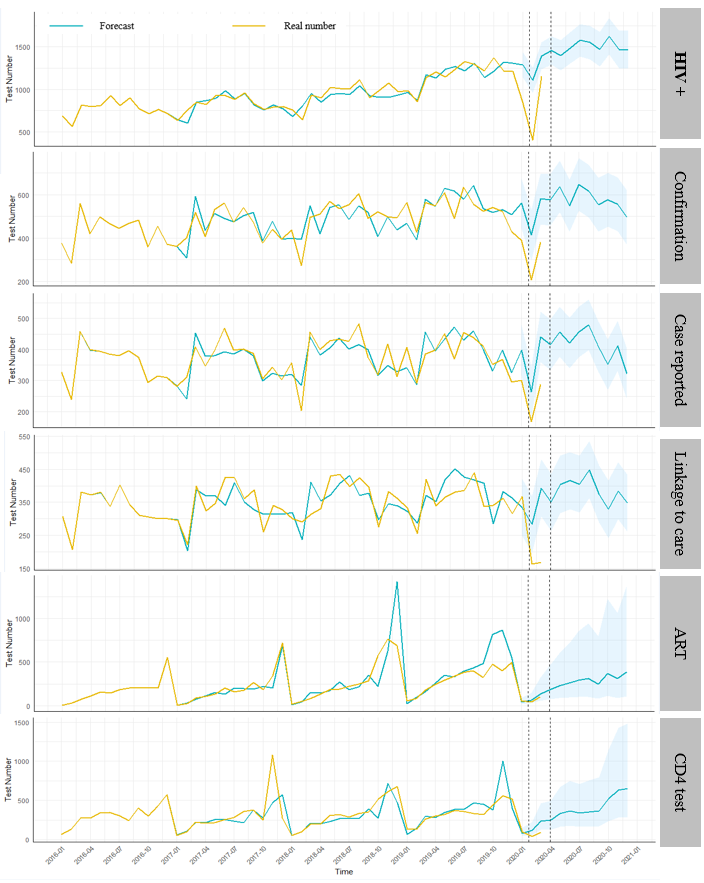


fig S1. Trend of forecast and real number of HIV from screen positive to ART during 2016 to 2020


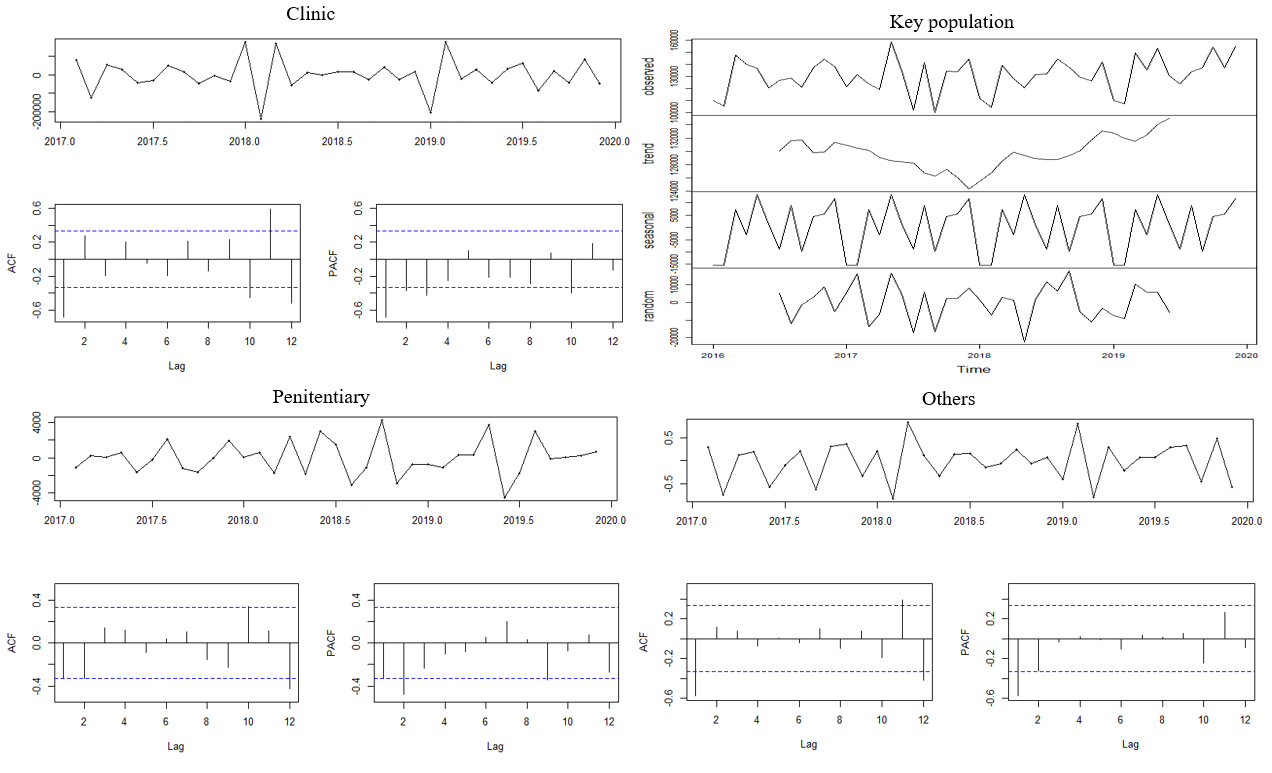


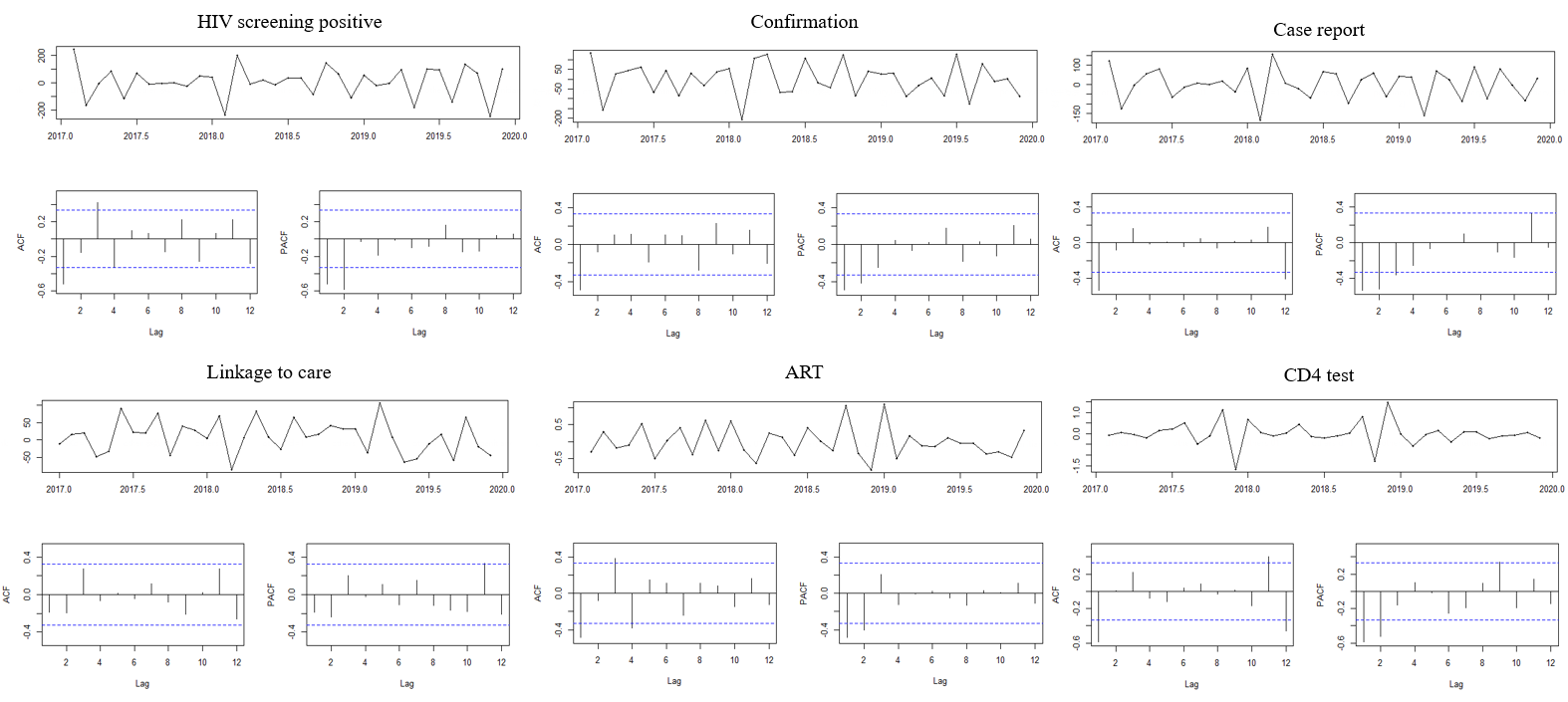


fig S2. Seasonal variation, autocorrelation function and partial autocorrelation function for the forecasting data


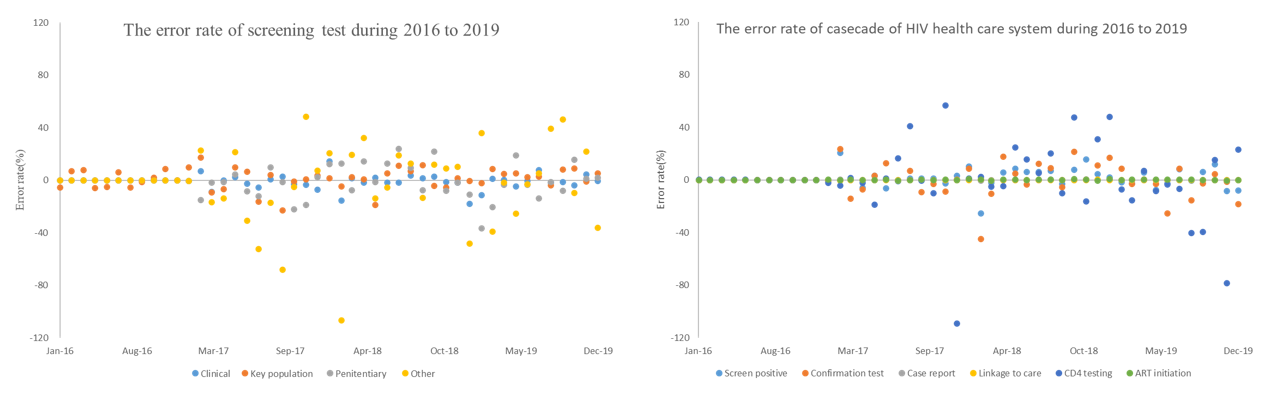


fig S3. The error rate in the cascade flow in HIV health care system during 2016 to 2019

table S1 Models parameters of the forecasting

| Variables | Parameters | ME | RMSE | MAE | MPE | MAPE | MASE | ACF1 | AIC | Ljung-Box | |
| --- | --- | --- | --- | --- | --- | --- | --- | --- | --- | --- | --- |
|  |  |  |  |  |  |  |  |  |  | Chi-square | P value |
| Clinic | ARIMA(0,1,1)(1,1,0)[12] | -5160.98 | 35082.33 | 21666.02 | -0.92 | 3.14 | 0.43 | -0.20 | 858.27 | 2.09 | 0.15 |
| Penitentiary | ARIMA(0,1,1) (1,1,0)[12] | 14.75 | 1025.12 | 699.72 | -0.92 | 7.67 | 0.22 | 0.15 | 611.41 | 1.14 | 0.29 |
| Others | ARIMA(0,1,1) (1,1,0)[12] | -514.12 | 11740.49 | 7886.26 | -2.69 | 17.37 | 0.52 | -0.09 | 774.30 | 0.45 | 0.50 |
| Screen positive | ARIMA(0,1,2) (0,1,0)[12] | 13.47 | 64.82 | 43.40 | 1.17 | 4.43 | 0.49 | 0.04 | 409.82 | 0.07 | 0.79 |
| Confirmation | ARIMA(0,1,1) (1,1,0)[12] | 2.37 | 50.45 | 34.37 | -0.13 | 7.25 | 0.46 | -0.07 | 394.19 | 0.25 | 0.61 |
| Case report | ARIMA(0,1,1) (1,1,0)[12] | 3.14 | 34.87 | 22.62 | 0.18 | 6.23 | 0.38 | -0.20 | 373.07 | 2.05 | 0.15 |
| Linkage to care | ARIMA(0,1,1) (1,1,0)[12] | -0.28 | 36.96 | 27.16 | -0.62 | 7.78 | 0.53 | -0.17 | 375.08 | 1.46 | 0.23 |
| Art | ARIMA(1,0,0) (0,1,0)[12] | 12.20 | 98.44 | 56.79 | -2.59 | 28.50 | 0.54 | 0.07 | 447.34 | 0.28 | 0.60 |
| CD4 | ARIMA(0,1,1) (1,1,0)[12] | 12.25 | 114.62 | 57.68 | -0.02 | 15.06 | 0.49 | -0.31 | 458.60 | 4.86 | 0.03 |
|  |  | Alpha | Beta | Gamma | - | - | - | - | AIC | - | - |
| Key-population | Holt-winter exponential smoothing | 0.01 | 0.01 | 0.0001 | - | - | - | - | 1104.08 | - | - |

-: Not available or necessary in this model.
